# Status of hepatic DNA methylome predetermines and modulates the severity of non-alcoholic fatty liver injury in mice

**DOI:** 10.1186/s12864-016-2617-2

**Published:** 2016-04-22

**Authors:** Volodymyr P. Tryndyak, Tao Han, James C. Fuscoe, Sharon A. Ross, Frederick A. Beland, Igor P. Pogribny

**Affiliations:** Division of Biochemical Toxicology, National Center for Toxicological Research, FDA, 3900 NCTR Rd, Jefferson, AR 72079 USA; Division of Systems Biology, National Center for Toxicological Research, FDA, Jefferson, AR USA; Division of Cancer Prevention, National Cancer Institute, Bethesda, MD USA

**Keywords:** DNA methylation, Non-alcoholic fatty liver injury, Susceptibility, Mouse, Inter-strain differences

## Abstract

**Background:**

Nonalcoholic fatty liver disease (NAFLD) is a major health problem and a leading cause of chronic liver disease in the United States and Western countries. In humans, genetic factors greatly influence individual susceptibility to NAFLD; nonetheless, the effect of inter-individual differences in the normal liver epigenome with regard to the susceptibility to NAFLD has not been determined.

**Results:**

In the present study, we investigated the association between the DNA methylation status in the livers of A/J and WSB/EiJ mice and the severity of NAFLD-associated liver injury. We demonstrate that A/J and WSB/EiJ mice, which are characterized by significant differences in the severity of liver injury induced by a choline- and folate-deficient (CFD) diet exhibit substantial differences in cytosine DNA methylation in their normal livers. Furthermore, feeding A/J and WSB/EiJ mice a CFD diet for 12 weeks resulted in different trends and changes in hepatic cytosine DNA methylation.

**Conclusion:**

Our findings indicate a primary role of hepatic DNA methylation in the pathogenesis of NAFLD and suggest that individual variations in DNA methylation across the genome may be a factor determining and influencing the vulnerability to NAFLD.

**Electronic supplementary material:**

The online version of this article (doi:10.1186/s12864-016-2617-2) contains supplementary material, which is available to authorized users.

## Background

Nonalcoholic fatty liver disease (NAFLD) is the most common cause of chronic liver disease and a major health problem in the United States and developed Western countries [1, 2]. The pathogenesis of NAFLD is complex and involves dysregulation of several interdependent molecular processes, including lipid metabolism, insulin resistance, immune response, inflammation, oxidative stress, and apoptosis [1]. NAFLD has two principal pathological phenotypic states – uncomplicated non-alcoholic fatty liver (NAFL) and non-alcoholic steatohepatitis (NASH), a progressive form of the disease characterized by lipid accumulation, inflammation, hepatocellular damage, and fibrosis in the liver [3, 4] that has a greater risk of developing life threatening liver-related complications, including hepatocellular carcinoma [5]. Importantly, in a recent study, McPherson et al. [6], using sequential paired liver biopsy samples from patients with histologically-confirmed NAFLD, demonstrated that 44 % of individuals diagnosed with NAFL progressed to NASH [6]. In light of this, it is critical to identify cohorts susceptible to NASH development at early stages of the disease progression; however, most of the current studies have focused on identifying the underlying molecular mechanisms of the pathogenesis and progression of NAFLD [7] and less attention has been given (i) to clarifying the determinants for the predisposition to the development of NASH in certain individuals, and (ii) how to identify these individuals. This is critical not only for identifying subpopulations sensitive to NAFLD, but also for its treatment and prevention, since there are no approved therapeutic drugs for the treatment of NASH [4, 8]. This indicates that there is an urgent need for a better understanding of the molecular mechanisms and processes associated with NASH development and for the discovery of determinants and biomarkers for enhancing the diagnosis and monitoring the progression to NASH [8].

The development of NAFLD and its progression to NASH, liver fibrosis, and cirrhosis has been associated with disease-specific genomic alterations, mainly with distinct cytosine DNA methylation and gene expression patterns [9–13]. Specifically, Murphy et al. demonstrated that differences in cytosine genomic DNA methylation can distinquish patients with NASH [10], and Pirola et al. reported that the level of cytosine mitochondrial DNA methylation is associated with the histological severity of NASH [11]. It is clear that the development and progression of NAFLD by itself can induce epigenetic alterations; however, epigenetic changes in the liver may also have an impact on disease development and progression. For instance, the results of comprehensive genome-wide association studies (GWAS) on patients with biopsy-confirmed NAFLD identified *PNPLA3* (patatin-like phospholipase domain containing 3) as a modifier of NAFLD outcome [14] and demonstrated that the genetic and epigenetic variations in the *PNPLA3* gene influence risk and features of NAFLD [14–16]. Additionally, the results of recent epigenome-wide association studies (EWAS) demonstrated that natural differences in DNA methylation caused by genetic variations are associated with metabolic traits and may contribute to complex disease etiology [17]; nonetheless, the influence of hepatic genome-wide epigenomic differences on susceptibility and severity to NAFLD has not been investigated.

Based on these considerations the goals of this study were (i) to determine whether or not the epigenome status in normal livers affects the severity of NAFLD-associated liver injury induced by feeding a choline- and folate-deficient (CFD) diet, and (ii) to investigate the role of specific DNA methylation changes in the pathogenesis of NAFLD liver injury. To achieve these goals, we investigated the status of hepatic cytosine DNA methylation and histone H3K4 trimethylation (H3K4me3) in A/J and WSB/EiJ mice fed a CFD diet. This diet consistently induces fat-related liver injury and is the most efficient and reproducible approach for inducing severe NAFLD-associated liver damage and fibrosis in mice that resembles the histopathological features of human NAFLD [18, 19]. More importantly, NAFLD induced by a CFD diet is an ideal model for studying the subgroup of NASH patients with histologically advanced NASH [19]. We demonstrate that A/J and WSB/EiJ mice, which exhibit significant variations in the severity of liver injury induced by a CFD diet [20], are characterized by substantial differences in cytosine DNA methylation in their normal livers, and that each strain of mice can be distinguished by its unique normal DNA methylation profile. More importantly, the severity of NAFLD-like liver injury in A/J and WSB/EiJ mice induced by the CFD diet was accompanied by different trends and changes in cytosine DNA methylation in their livers. This suggests that the status of hepatic DNA methylome may influence individual susceptibility to NAFLD. Furthermore, our findings indicate that the evaluation of DNA methylation may be helpful in diagnosis and monitoring the severity of NAFLD-associated liver injury.

## Results

### Status of cytosine DNA methylation and histone H3K4 trimethylation in the livers of control A/J and WSB/EiJ mice

**Fig. 1:**
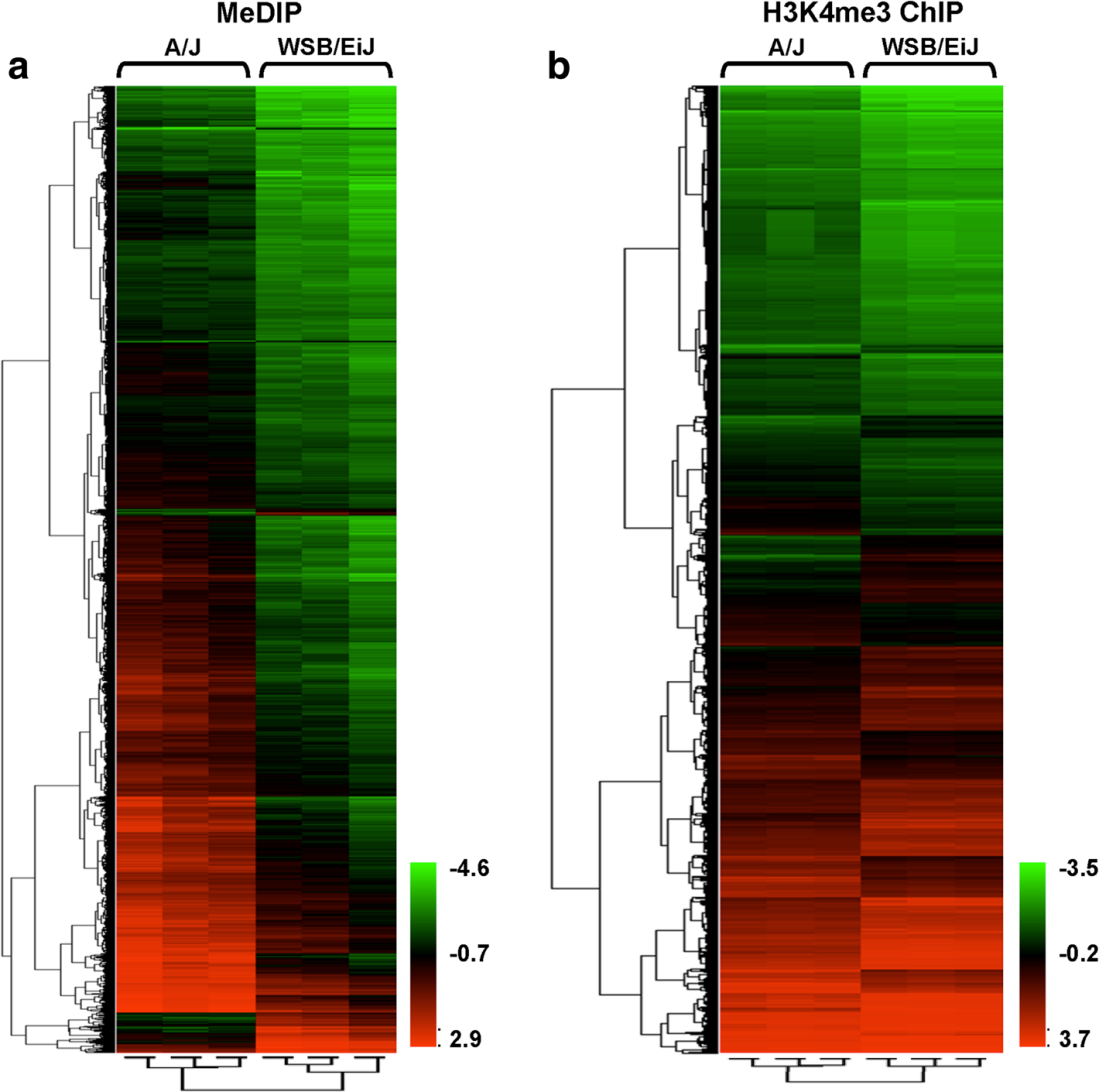
DNA methylation and H3K4me3 in the normal livers of A/J and WSB/EiJ mice. **a** MeDIP microarray analysis of CpG island methylation in the normal livers of A/J and WSB/EiJ mice. **b** ChIP-on-chip analysis of H3K4me3 in the normal livers of A/J and WSB/EiL mice. Heat map illustrating significant differences in hepatic CpG island methylation between A/J and WSB/EiJ control mice. Unsupervised hierarchical clustering analysis was performed using one-way ANOVA with *p* value cut-off at 0.05. The color bar identifies high-methylated or high H3K4me3-enriched (*red*) and low-methylated or low H3K4me3-enriched (*green*) CpG islands

The status of cytosine DNA and histone H3K4 trimethylation, a well-established major histone transcription-activating mark [21], in the normal livers of A/J mice, a strain characterized by a mild NAFLD-like liver injury, and WSB/EiJ mice, a strain that exhibits severe NASH-like liver injury induced by choline and folate deficiency [20], was evaluated by methylated DNA immunoprecipitation (meDIP) and chromatin immunoprecipitation (ChIP) assays in combination with Agilent Mouse 2 × 105 K CpG Island Microarrays that cover 16,030 CpG islands of the mouse genome. Unsupervised hierarchical clustering of the CpG methylation and histone H3K4me3 data showed that A/J and WSB/EiJ mouse strains could be distinguished by their hepatic CpG island methylation and histone H3K4 methylation profiles (Fig. 1). A further detailed analysis of the microarray CpG island methylation data revealed substantial differences in cytosine DNA methylation patterns in the livers of control A/J and WSB/EiJ mice (Table 1). Specifically, the proportion of hypermethylated CpG islands in genomic DNA isolated from the livers of control A/J mice and located in the promoter, gene body, downstream, divergent promoter, and total gene regions was significantly greater than in WSB/EiJ mice (Table 1). In contrast, DNA isolated from the liver of control WSB/EiJ mice possessed a higher proportion of unmethylated CpG islands than A/J mice (Table 1). In contrast to hepatic CpG island methylation, the proportion of histone H3K4 trimethylation did not differ between the two strains in the downstream, divergent, and total gene regions, although there was a significant increase in the proportion of histone H3K4 trimethylation in the promoter region of A/J mice and in the gene body region of WSB/EiJ mice (Table 1).

**Table 1:** Number of differentially methylated and H3K4me3-enriched CpG islands (CGIs) in hepatic DNA of control A/J and WSB/EiJ mice

Location	MeDIP				ChIP-on-chip			
	Strain	Number of hypo- or hyper-methylated CGIs^a^	Proportion hyper-methylated CGIs	z-test^c^	Strain	Number of CGIs with decreased or increased H3K4me3 enrichment^b^	Proportion with increased H3K4me3 enrichment	z-test^c^
Promoter	A/J	1254	0.40	*P* < 0.001	A/J	2578	0.47	*P* < 0.001
	WSB/EiJ	1254	0.10		WSB/EiJ	2578	0.41	
Gene Body	A/J	2371	0.52	*P* < 0.001	A/J	4828	0.52	*P* = 0.015
	WSB/EiJ	2371	0.14		WSB/EiJ	4828	0.54	
Downstream	A/J	128	0.57	*P* < 0.001	A/J	201	0.70	*P* > 0.05
	WSB/EiJ	128	0.11		WSB/EiJ	201	0.66	
Divergent promoter	A/J	147	0.37	*P* < 0.001	A/J	341	0.35	*P* > 0.05
	WSB/EiJ	147	0.07		WSB/EiJ	341	0.30	
Total	A/J	3900	0.48	*P* < 0.001	A/J	7948	0.50	*P* > 0.05
	WSB/EiJ	3900	0.12		WSB/EiJ	7948	0.49	

^a^CGI fragments with a negative Z score and *p* < 0.05 were considered being significantly hypomethylated, and CGI fragments with a positive Z score and *p* < 0.05 were considered being significantly hypermethylated

^b^The H3K4me3 enrichment was considered to be significant if the CGI fragments ratio IP/input DNA p value was < 0.05

^c^Comparisons in the extent of hypermethylation and H3K4me3 enrichment between A/J and WSB/EiJ mice were conducted by one-tailed z-tests

### Effect of the CFD diet on CpG island DNA methylation in the livers of A/J and WSB/EiJ mice

**Fig. 2:**
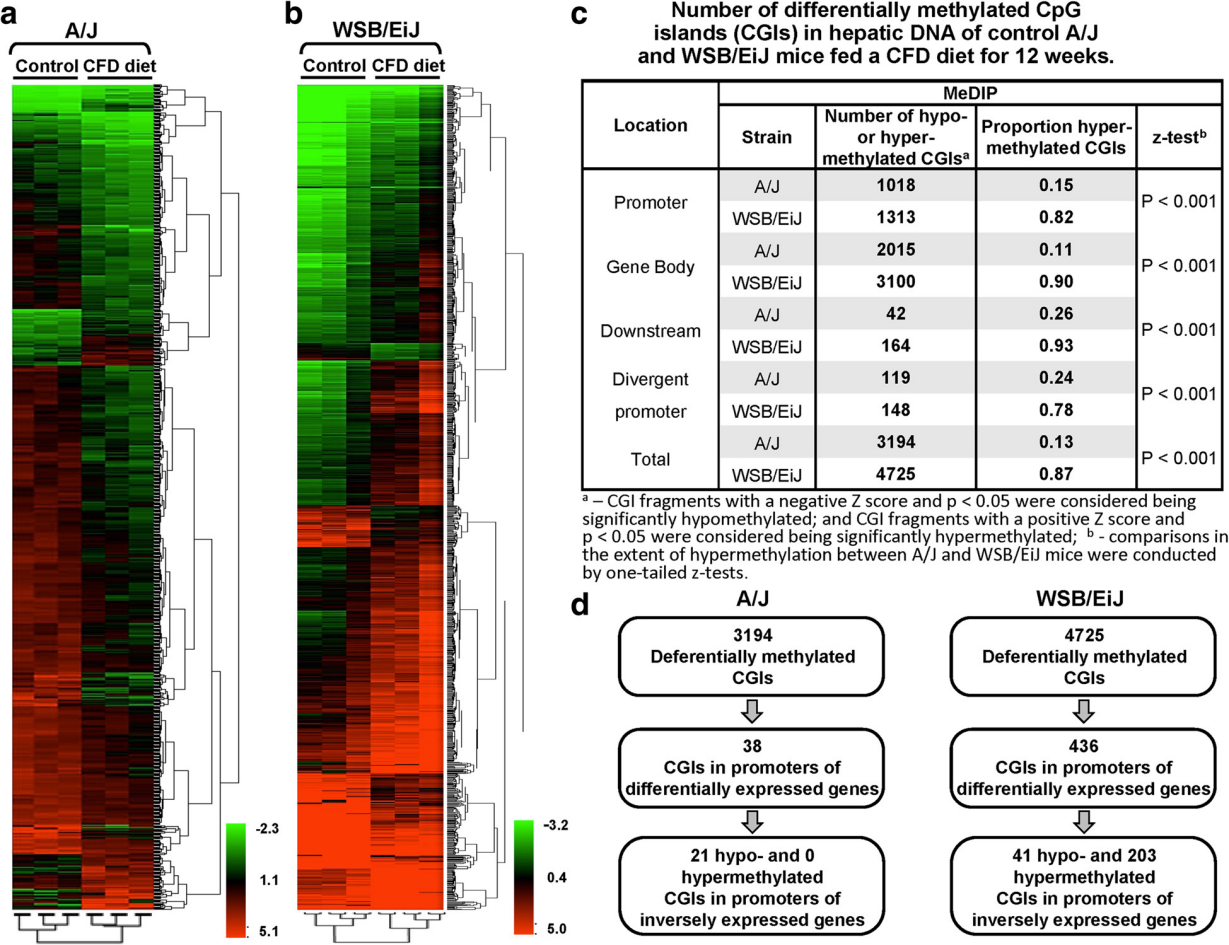
MeDIP microarray analysis of CpG island methylation changes in the livers of A/J and WSB/EiJ mice fed a choline- and folate-deficient diet. Heat map illustrating significant differences in hepatic CpG island methylation between A/J (**a**) and WSB/EiJ (**b**) mice fed the CFD diet. Unsupervised hierarchical clustering analysis was performed using one-way ANOVA, with *p* value cut-off at 0.05. The color bar identifies high-methylated (*red*) and low-methylated (*green*) genes. **c** Table showing the number of differentially methylated CpG islands in hepatic DNA in A/J and WSB/EiJ mice fed a control or CFD diet. Z-scores were calculated with Agilent Genomic Workbench Light. **d** Algorithm of analysis of inversely differentially methylated and expressed genes in the livers of A/J and WSB/EiJ mice fed a CFD diet

To elucidate further the role of cytosine DNA methylation and histone H3K4 trimethylation in the susceptibility to NAFLD and the association between these epigenetic marks and histopathological severity and features of NAFLD-associated liver injury, the status of CpG island methylation and histone H3K4me3 in the livers of A/J and WSB/EiJ mice fed a CFD diet was investigated. Figure 2 shows that feeding the CFD diet resulted in marked strain-dependent changes in CpG island methylation. Unsupervised hierarchical clustering of the CpG island methylation data showed two important findings: first, A/J and WSB/EiJ mice fed the CFD diet could be distinguished from mice on the control diet within each strain (Fig. 2a and b); and second, A/J and WSB/EiJ mice fed the CFD diet exhibited different trends and patterns in hepatic cytosine DNA methylation that were associated with the severity of NALD-like liver injury.

To identify differentially methylated CpG islands between the control and CFD diet-fed groups within each strain, a *t*-test with Benjamini-Hochberg correction, *p* < 0.05 was applied. A total of 3194 and 4725 CpG islands was found to be differentially methylated, respectively, in the livers of A/J and WSB/EiJ mice fed the CFD diet (Fig. 2c). Further analysis of these CpG island methylation changes demonstrated that feeding the CFD diet caused marked differences in cytosine DNA methylation between the two strains. Specifically, feeding the CFD diet resulted in a marked increase in the proportion of hypomethylated CpG islands in the livers of A/J mice, while in the livers of WSB/EiJ mice, there was a marked increase in the proportion of hypermethylated CpG islands (Fig. 2c). The number of hypomethylated genes in A/J CFD diet-fed mice corresponded to a greater number of up-regulated genes, whereas the number of hypermethylated genes in WSB/EiJ mice was associated with a greater number of down-regulated genes (GEO accession number GSE62362) [22]. Furthermore, pairing DNA methylation and transcriptomic analyses of differentially methylated genes identified 21 hypomethylated and up-regulated CpG island promoter containing genes, whereas not a single hypermethylated gene that correlated inversely with gene expression was found in A/J mice (Fig. 2d). An opposite trend of changes was found in WSB/EiJ mice, where 203 CpG island promoter containing genes were hypermethylated and correlated inversely with gene expression and only 41 genes were hypomethylated and up-regulated (Fig. 2d, Additional file 1: Table S1).

In contrast to DNA methylation changes in the livers of A/J and WSB/EiJ mice fed the CFD diet, only 552 and 1366 CpG islands, respectively, were found to have differential enrichment by histone H3K4me3 (Additional file 2: Figure S1). Importantly, no major differences in histone H3K4me3 were found between A/J and WSB/EiJ mice fed the CFD diet, although the promoter region was slightly enriched in WSB/EiJ mice compared to A/J mice, while the opposite trend occurred in the gene body region.

### CpG methylation changes are associated with key NAFLD pathological pathways

**Fig. 3:**
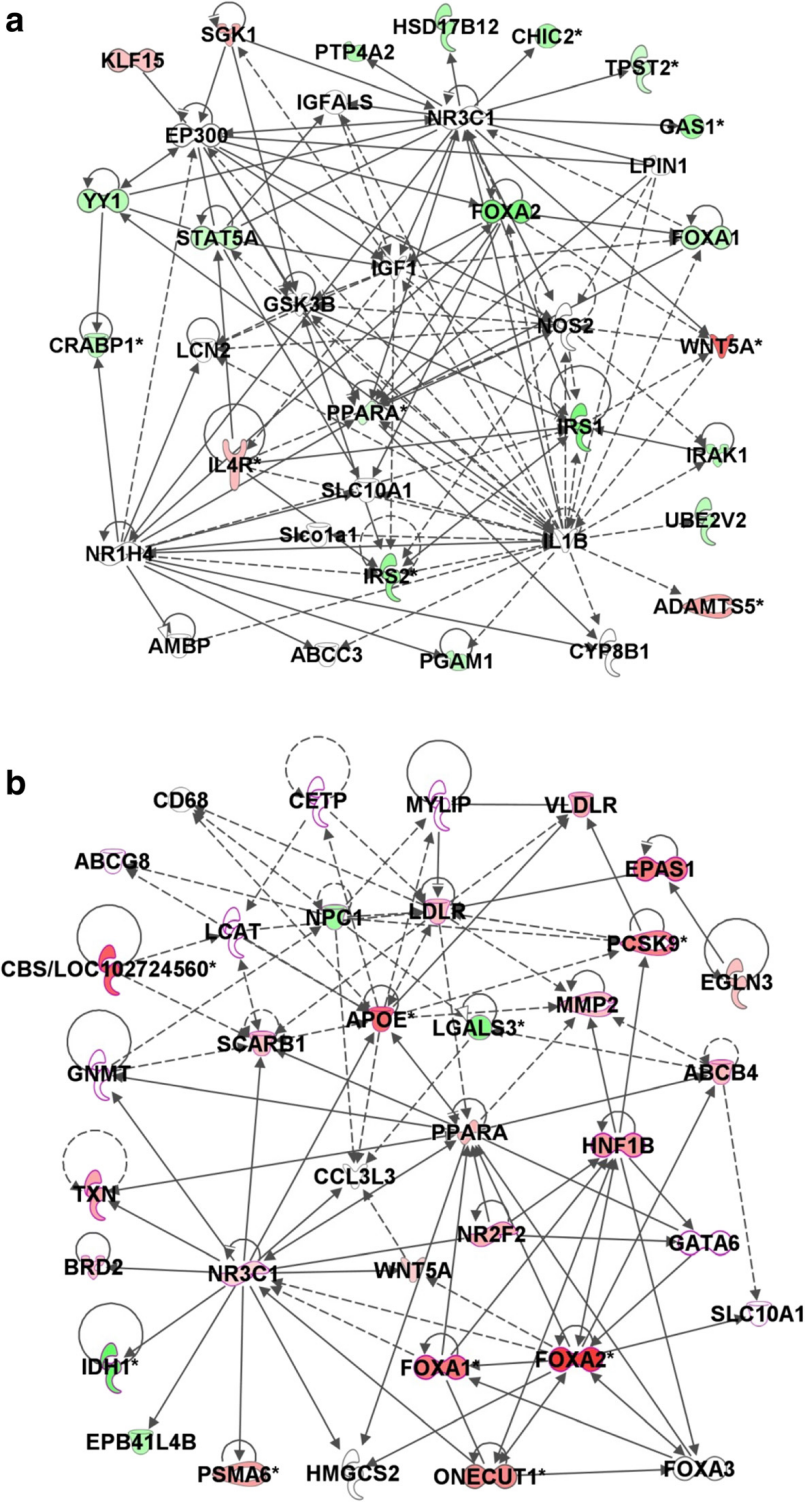
Molecular network interactions between differentially methylated genes in the livers of A/J (**a**) and WSB/EiJ (**b**) mice significantly different between control and CFD diets. The Ingenuity Pathway Analysis database was used to determine and visualize molecular pathways enriched by the significant differentially methylated CpG islands of genes. Hypermethylated genes are marked in red color and hypomethylated genes in green color

**Fig. 4:**
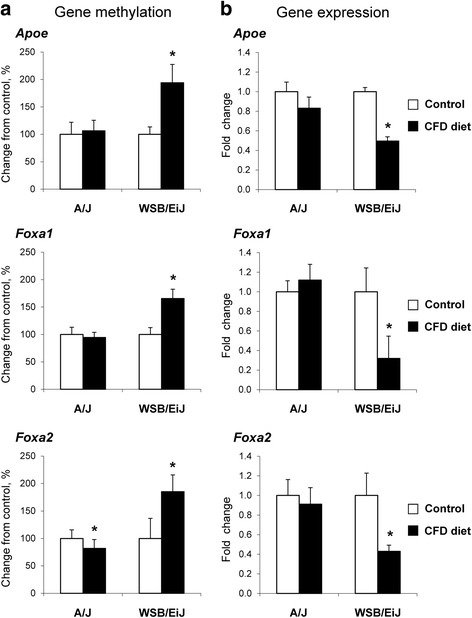
Status of promoter cytosine DNA methylation and gene expression of *Apoe*, *Foxa1*, and *Foxa2* in the livers of mice fed a control or choline- and folate-deficient diet. **a** Gene-specific cytosine DNA methylation was determined by using MeDIP DNA coupled with real time PCR with primers specific to promoter regions of *Apoe*, *Foxa1*, and *Foxa2* genes in the livers of control and CFD diet-fed groups. The results are presented as the percent change of promoter methylation of each gene in the livers of mice fed the CFD diet relative to that in control groups, which were assigned a 100 %. **b** qRT-PCR analysis of *Apoe*, *Foxa1*, and *Foxa2* gene expression in the livers of mice fed a control or CFD diet. The results are presented as the average fold change in the expression of each gene in the livers of mice fed the CFD diet relative to that in control groups, which were assigned a value 1. White bars – control mice, black bars – mice fed the CFD diet. * - Significantly different from the strain-matched control mice (mean ± SD, *n* = 5, fold change > 2.0, *p* <0.05)

To evaluate further whether or not changes in CpG island methylation are involved in the deregulation of key physiological pathways associated with NAFLD development and determine if differences in cytosine DNA methylation can explain the difference in severity of NAFLD-like liver injury in A/J and WSB/EiJ mice fed the CFD diet, a pathway enrichment analysis of the differentially methylated CpG island genes was performed. Analysis of differentially methylated CpG islands in the livers of WSB/EiJ mice fed the CFD diet demonstrated a strong enrichment in genes associated with cell proliferation and organismal injury and abnormalities; and lipid metabolism, molecular transport, and small-molecule biochemistry networks involved in the NAFLD pathogenesis (Table 2). In the livers of A/J mice, the top molecular pathways enriched by differentially methylated genes were pathways associated with cell morphology, tissue development, and carbohydrate metabolism; organismal injury and abnormalities, and cell death and survival; lipid metabolism, molecular transport, and small-molecule biochemistry (Table 2). Although some of the differentially methylated CpG island networks were common to both strains of mice (e.g., lipid metabolism, molecular transport, and small-molecule biochemistry), the major difference between the two strains was the extensive hypermethylation in WSB/EiJ mice and extensive hypomethylation in A/J mice. Importantly, these changes in cytosine DNA methylation corresponded with the gene expression in CFD diet-fed A/J and WSB/EiJ mice. For example, Fig. 3 shows that the majority of the genes in the metabolic PPAR-regulated network were hypomethylated in the livers of A/J mice fed the CFD diet (Fig. 3a) and hypermethylated in the livers of WSB/EiJ mice (Fig. 3b). Previously, we demonstrated that the metabolic PPAR-regulated network, a major network affected by CFD diets [22], was activated in A/J mice and down-regulated in WSB/EiJ mice [22]. In order to confirm this observation, we evaluated the status of gene-specific cytosine DNA methylation and gene expression of *Apoe*, *Foxa1*, and *Foxa2*, three of the most differentially-methylated genes of the PPAR-regulated network. Figure 4 shows that the extent of *Apoe*, *Foxa1*, and *Foxa2* promoter methylation was increased by 94, 66, and 85 %, respectively, in the livers of WSB/EiJ mice fed the CFD diet (Fig. 4a), but was not changed, except for a minor decrease in methylation of the *Foxa2* gene, in the livers of A/J mice (Fig. 4a). Importantly, hypermethylation of *Apoe*, *Foxa1*, and *Foxa2* in the livers of WSB/EiJ mice was accompanied by a reduced expression of these genes (Fig. 4b).

**Table 2:**
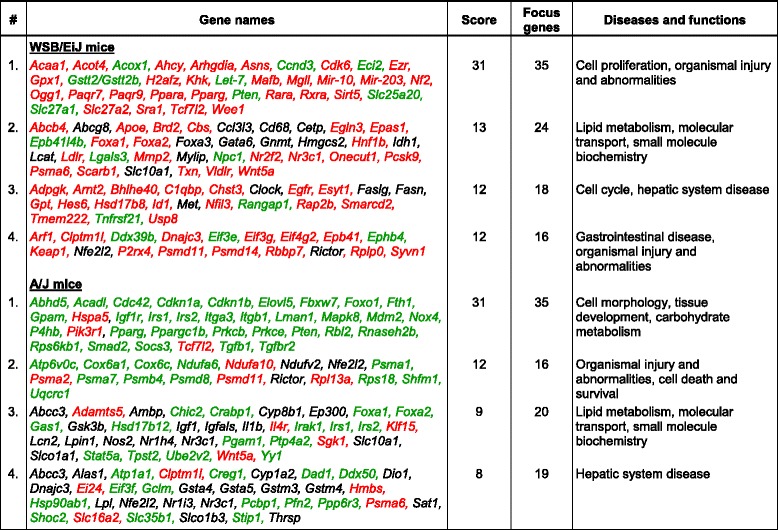
Top molecular networks of differentially methylated CpG-island-containing genes in the livers of A/J and WSB/EiJ mice fed the CFD diet

Genes in green color – hypomethylated; red color – hypermethylated; and black color – no change in methylation

### Effect of the CFD diet on DNA methyltransferases

**Fig. 5:**
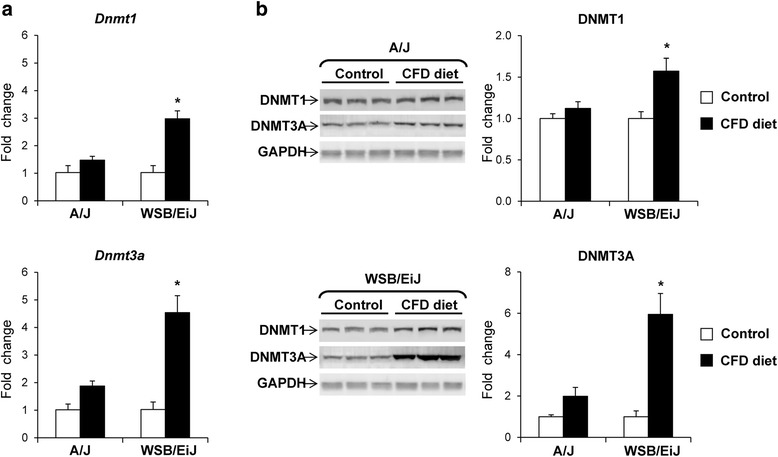
Expression of DNA methyltransferase genes in the livers of mice fed a CFD diet. qRT-PCR analysis of *Dnmt1* and *Dnmt3a* mRNA transcripts (**a**) and Western blot analysis of DNMT1 and DNMT3A proteins (**b**). The results are presented as the average fold change in the expression of each gene in the livers of mice fed the CFD diet relative to that in control groups, which were assigned a value 1. White bars – control mice, black bars – mice fed the CFD diet. * - Significantly different from the strain-matched control mice (mean ± SD, *n* = 5, fold change >2.0, *p* < 0.05)

To evaluate mechanisms that may be associated with changes in CpG island methylation induced by the CFD diet, the expression of the maintenance DNA methyltransferase 1 (*Dnmt1*) and *de novo* DNA methyltransferases *Dnmt3a* and *Dnmt3b* genes was examined. Figure 5 shows that the expression of *Dnmt1* and, especially, *Dnmt3a* genes and the level of DNMT1 and DNMT3A proteins were significantly increased in the livers of WSB/EiJ mice fed the CFD diet, whereas feeding the CFD diet did not change the expression of *Dnmt1* and *Dnmt3a* genes and DNMT1 and DNMT3A protein levels in the livers of A/J mice. In contrast, the levels of *Dnmt3b* mRNA and DNMT3B protein in the livers of CFD diet fed A/J and WSB/EiJ mice did not differ from those in the control mice (data not shown).

## Discussion

Accumulated evidence in the field of genomic research has indicated clearly that inter-individual genetic variations in hepatic lipid metabolism, oxidative stress response, and proinflamamatory and fibrogenic pathways greatly influence individual susceptibility, severity, and pathological features of NAFLD in humans [23–27]; however, these variants account for only a small fraction of the disease. In contrast, epigenetically-associated differences in chromatin structure and gene expression may contribute to the entire spectrum of NAFLD-associated phenotypic alterations. Previously, we demonstrated that feeding mice a CFD diet induced NAFLD-like liver injury that was accompanied by substantial hepatic DNA methylation changes, the magnitude of which correlated with the severity of liver injury [28]. Importantly, similar findings have been reported by Murphy et al*.* and Pirola et al., who demonstrated that differences in cytosine methylation of hepatic genomic and mitochondrial DNA were associated with the severity of morphological changes in patients with a biopsy-confirmed NAFLD [10, 11].

In this study, we investigated whether or not variations in normal hepatic cytosine DNA methylation affected the susceptibility to NAFLD and were associated with the severity of NAFLD-associated liver injury. Our results demonstrate that A/J mice, characterized by a mild NAFLD-like liver injury, and WSB/EiJ mice, characterized by a severe NASH-like liver injury [21], exhibited substantial differences in cytosine DNA methylation in their normal livers, and that each strain of mice could be distinguished by its unique DNA methylation profile. Specifically, A/J mice were characterized by a greater extent of CpG island methylation compared to NASH injury-prone WSB/EiJ mice. This suggests that the status of the DNA methylome of normal liver may determine individual susceptibility to NAFLD. These findings are in good agreement with a recent report by Orozco et al. [17], who profiled the DNA methylation status of livers from 90 inbred mouse strains and demonstrated that a natural variation in CpG methylation levels is associated with metabolic traits and may contribute to complex disease etiology [17]. Importantly, our results demonstrate that mice with a lower hepatic DNA methylation phenotype are more prone to liver injury.

Another major finding of this study is that differences in CpG island-specific methylation responses, either hypomethylation or hypermethylation, to feeding the CFD diet were associated with the magnitude of NAFLD-associated injury. The responses depended on the initial status of CpG island methylation in the normal livers. For example, in A/J mice, a strain that exhibited a more methylated phenotype in the normal livers and a mild NAFLD-like liver injury, the CFD diet caused hypomethylative changes in CpG islands, whereas in WSB/EiJ mice, which are characterized by a less methylated original hepatic phenotype and severe NASH-like liver injury, feeding the CFD diet caused profound hypermethylative changes driven by up-regulation of DNMT1 and DNMT3A and concurrent inhibition of gene expression. This was further demonstrated by methylation/expression changes of *Apoe* (apolipoprotein E), *Foxa1* (forkhead box A1), and *Foxa2* (forkhead box A2), genes essential in controlling fundamental metabolic pathways and homeostasis in the liver, which were among the top (in terms of both significance and fold change) differentially methylated/expressed transcripts. These findings are in a good agreement with the previous report [11] that showed a significant up-regulation of *DNMT1* and marked hypermethylation and down-regulation of the mitochondrially encoded NADH-dehydrogenase 6 (*MT-ND6*) gene in NASH patients.

Several studies have demonstrated that down-regulation of the FOXA subfamily members, FOXA1 and FOXA3, play an important role in the pathogenesis and progression of NAFLD in both humans and experimental animals [29–31]. Similarly, a recent study by Schierwagen et al. demonstrated that feeding a Western diet induced metabolic syndrome and development of NASH in *Apoe*-deficient mice [32]. Furthermore, Benet et al. showed that treatment of human hepatocytes in vitro or rats in vivo with steatotic drugs repressed *Foxa1* expression [33]. Mechanistically, the reduction of *Foxa1* expression has been associated with several pathological phenotypic features of NAFLD, including induction of triglyceride synthesis and accumulation, inhibition of fatty acid β-oxidation, and homocysteinemia [29–31]. These effects were attributed mainly to down-regulation of a number of FOXA1-controlled genes in the lipid and one-carbon metabolism networks. Several mechanisms might account for the inhibition of *Foxa1* in NAFLD, including direct repression of *Foxa1* via deregulation of the protein kinase C pathway [29] and down-regulation of CCAAT/enhancer binding protein beta (C/EBPβ) [34] or an indirect mechanism via inhibition of the TGF-beta signaling pathway by hepatocyte nuclear factor 6 (HNF-6) [35] or deregulation of miR-122/FOXA1/HNF4a feedback loop [36]. In the present study, we describe another mechanism of *Foxa1* inhibition in NAFLD development and progression that is mediated by gene-specific cytosine DNA hypermethylation and may be attributed to a profound over-expression of DNA methyltransferases in the livers of NAFLD-prone WSB/EiJ mice.

## Conclusions

In conclusion, the results of the present study demonstrate a primary role of DNA methylation in the pathogenesis of NAFLD and emphasize the importance of the between-individual variation in DNA methylation across the genome as a factor determining the NAFLD vulnerability. Furthermore, our study suggests that the evaluation of the DNA methylation status may be critical for the assessment of the disease susceptibility and its severity. Additionally, considering the potential reversibility of epigenetic changes, our study suggests that modulation of DNA methylation may modify the severity of NAFLD-associated liver injury.

## Methods

### Animals and experimental design

Male A/J and WSB/EiJ mice (6 weeks of age) were obtained from the Jackson Laboratory (Bar Harbor, ME, USA). These strains were selected because they exhibited significant differences in the extent of NAFLD-associated liver injury induced by a methyl-donor-deficient diet [22]. The in-life portion of this study, diet description, mouse treatment, tissue collection, and results of histopathological and clinical chemistry analyses are described in detail in Tryndyak et al. [22]. All experimental procedures were reviewed and approved by the National Center for Toxicological Research Animal Care and Use Committee.

### Methylated DNA immunoprecipitation microarray analysis

Genomic DNA was isolated from mouse liver tissues using DNeasy Blood and Tissue kits (Qiagen, Valencia, CA, USA). Methylated DNA immunoprecipitation (MeDIP) was performed with Methyl Miner Methylated DNA Enrichment kits (Life Technologies, Grand Island, NY, USA) according to the manufacturer’s instructions. Briefly, 5 μg of genomic DNA was randomly sheared by sonication to an average length of 0.2–1.0 kb and divided into immunoprecipitated and input portions. DNA from the immunoprecipitated portions was incubated for 1 h at room temperature with MBD-Biotin protein coupled to M280 Streptavidin Dynabeads. The captured methylated DNA was eluted as a single fraction using a high-salt elution and precipitated with ethanol. The immunoprecipitated DNA and input DNA pellets were then dissolved in 30 μL of water for labeling. Immunoprecipitated DNA and input DNA samples were labeled with cyanine 3-dUTP and cyanine 5-dUTP, respectively, using an Agilent Genomic DNA Labeling Kit Plus (Agilent Technologies, Santa Clara, CA, USA). The labeled DNA samples were purified using Amicon Ultra-0.5 30 kDa columns (Millipore, Billerica, MA, USA) and eluted with 22 μL of 10 mM Tris, 1 mM EDTA buffer, pH 8.0. The labeled immunoprecipitated DNA and input DNA were co-hybridized to Agilent Mouse 2 × 105 K CpG Island Microarrays, containing ~97,652 oligonucleotide probes covering 16,030 CpG islands, according to the manufacturer’s protocol. All DNA samples from control and CFD diet-fed A/J and WSB/EiJ mice in the MeDIP microarray experiment were processed simultaneously. Microarrays were scanned on an Agilent DNA microarray scanner and the resulting images were analyzed with Agilent Feature Extraction Software (Version 10.7.3). The combined Z-score for each probe was calculated with Agilent Genomic Workbench Light (Version 6.0). The data files were loaded into the ArrayTrack database [37] for further statistical analyses.

### Histone H_3_K_4_me3 chromatin immunoprecipitation microarray analysis

Formaldehyde crosslinking and chromatin immunoprecipitation (ChIP) assays were performed with primary antibodies against histone H3K4me3 (EMD Millipore, Billerica, MA, USA) using a Magna ChIP™ A chromatin immunoprecipitation kit (EMD Millipore). Sheared by sonication and purified, immunoprecipitated DNA and input DNA were amplified by a GenomePlex® Complete Whole Genome Amplification kit (Sigma-Aldrich, St. Louis, MO, USA). Amplified immunoprecipitated DNA and input DNA samples were labeled with cyanine 3-dUTP and cyanine 5-dUTP, respectively, using an Agilent Genomic DNA Labeling Kit Plus (Agilent Technologies, Santa Clara, CA, USA). The labeled DNA samples were purified using Amicon Ultra-0.5 30 kDa columns (Millipore, Billerica, MA, USA) and eluted with 22 μL of 10 mM Tris, 1 mM EDTA buffer, pH 8.0. The labeled immunoprecipitated DNA and input DNA were co-hybridized to Agilent Mouse 2 × 105 K CpG Island Microarrays according to the manufacturer’s protocol. Microarrays were scanned on an Agilent DNA microarray scanner and the resulting images were analyzed with Agilent Feature Extraction Software (Version 10.7.3). The data files were loaded into the ArrayTrack database [37] for Lowess normalization, calculation of the ratio of immunoprecipitated/input DNA, and further statistical analyses.

### Functional analysis of significant differentially methylated and H_3_K_4_me3-enriched genes

Ingenuity Pathway Analysis (IPA) software (Qiagen, Redwood City, CA, USA) was used to determine molecular pathways and networks that were enriched for significant differentially methylated or H3K4me3-enriched genes identified from *t* test analyses. Significance values was calculated based on a right-tailed Fisher’s exact test that determined whether a pathway was overrepresented by calculating whether the genes in a given pathway were enriched within the data set compared to all genes on the array; a *p* < 0.05 was selected as the cutoff for significance based on IPA threshold recommendations. Only those pathways with a *p* value below the threshold of *p* < 0.05 and having >3 representative genes in the data set were considered significant.

### RNA extraction and quantitative reverse transcription-PCR (qRT-PCR)

Total RNA was extracted from liver tissue using RNeasy Mini kits (Qiagen) according to the manufacturer’s instructions. Total RNA (2 μg) was reverse transcribed using random primers and High Capacity cDNA Reverse Transcription kits (Life Technologies) according to the manufacturer’s protocol. The following gene expression assays (Life Technologies) were used for quantitative reverse-transcription polymerase chain reaction (qRT-PCR): *Dnmt1* (Mm01151063_m1); *Dnmt3a* (Mm00432881_m1), and *Dnmt3b* (Mm01240113_m1). Reactions were performed in a 96-well assay format using a QuantStudio™ 7 Flex Real-Time PCR System (Life Technologies). Each plate contained one experimental gene and a housekeeping gene [*Gapdh* (Mm99999915_g1)], and all samples were plated in duplicate. The relative amount of each mRNA transcript was determined using the 2^−ΔΔCt^ method [38] and the results are presented as fold change. Values of fold change > 2.0 and *p* < 0.05 were considered significant.

### Western blotting

The level of DNMT proteins was determined by Western blot analysis using primary antibodies against DNMT1, DNMT3A, and DNMT3B (Abcam, Cambridge, MA, USA) as described in Tryndyak et al. [22]. Equal protein loading was confirmed by immunostaining against GAPDH (Abcam).

### Statistical analyses

Results are presented as mean ± S.D. Changes in gene specific methylation and expression were assessed by t-tests. Comparisons in the extent of hypermethylation and H3K4me3 enrichment were conducted by one-tailed z-tests. *P*-values < 0.05 were considered significant.

### Declarations

The views expressed in this paper do not necessarily represent those of the U.S. Food and Drug Administration.

### Ethics approval and consent to participate

All experimental procedures involving animals were reviewed and approved by the National Center for Toxicological Research Animal Care and Use Committee.

### Consent for publication

Not applicable

### Availability of data and materials

The datasets supporting the conclusion of this article are available in the Gene Expression Omnibus repository (GEO accession number GSE62362; http://www.ncbi.nlm.nih.gov/geo/query/acc.cgi?acc=GSE62362) and included within the article and its additional files.

## Additional files

Additional file 1: Table S1. Differentially methylated CpG island (CGI) in promoters of differentially expressed genes in the livers of WSB/EiJ mice fed a CFD diet for 12 weeks. (DOC 298 kb) Additional file 2: Figure S1. H3K4me3 ChIP-on-chip analysis in the livers of A/J and WSB/EiJ mice fed a choline- and folate-deficient diet. Heat map illustrating significant differences in hepatic H3K4me3-enrichment of CpG islands between A/J (**a**) and WSB/EiJ (**b**) mice fed the CFD diet. Unsupervised hierarchical clustering analysis was performed using one-way ANOVA with p value cut-off at 0.05. The color bar identifies high-H3K4me3-enriched (red) and low-H3K4me3-enriched (green) genes. (**c**) Table showing number of differentially H3K4me3-enriched CpG islands in hepatic DNA in A/J and WSB/EiJ mice fed a control or CFD diet. Ratio IP/input DNA was calculated using ArrayTrack application. (TIFF 1501 kb)

## Abbreviations

CFD: choline- and folate-deficient
GWAS: genome-wide association studies
MeDIP: methylated DNA immunoprecipitation
NAFLD: nonalcoholic fatty liver disease
NASH: nonalcoholic steatohepatitis
qRT-PCR: quantitative reverse-transcription polymerase chain reaction

## References

[1] Cohen JC, Horton JD, Hobbs HH (2011). Human fatty liver disease: old questions and new insights. Science.

[2] Rinella ME (2015). Nonalcoholic fatty liver disease: a systematic review. JAMA.

[3] Demir M, Lang S, Steffen H-M (2015). Nonalcoholic fatty liver disease - current status and future directions. J Dig Dis.

[4] Sanyal AJ (2015). Novel therapeutic targets for steatohepatitis. Clin Res Hepatol Gastroenterol.

[5] Baffy G, Brunt EM, Caldwell SH (2012). Hepatocellular carcinoma in non-alcoholic fatty liver disease: an emerging menace. J Hepatol.

[6] McPherson S, Hardy T, Henderson E, Burt AD, Day CP, Anstee QM (2015). Evidence of NAFLD progression from steatosis to fibrosing-steatohepatitis using paired biopsies: implications for prognosis and clinical management. J Hepatol.

[7] Peverill W, Powell LW, Skoien R (2014). Evolving concepts in the pathogenesis of NASH: beyond steatosis and inflammation. Int J Mol Sci.

[8] Ratziu V, Goodman Z, Sanyal A (2015). Current efforts and trends in the treatment of NASH. J Hepatol.

[9] Ahrens M, Ammerpohl O, von Schönfels W, Kolarova J, Bens S, Itzel T (2013). DNA methylation analysis in nonalcoholic fatty liver disease suggests distinct disease-specific and remodeling signatures after bariatric surgery. Cell Metab.

[10] Murphy SK, Yang H, Moylan CA, Pang H, Dellinger A, Abdelmalek MF (2013). Relationship between methylome and transcriptome in patients with nonalcoholic fatty liver disease. Gastroenterology.

[11] Pirola CJ, Gianotti TF, Burgueño AL, Rey-Funes M, Loidl CF, Mallardi P (2013). Epigenetic modification of liver mitochondrial DNA is associated with histological severity of nonalcoholic fatty liver disease. Gut.

[12] Zeybel M, Hardy T, Robinson SM, Fox C, Anstee QM, Ness T (2015). Differential DNA methylation of genes involved in fibrosis progression in non-alcoholic fatty liver disease and alcoholic liver disease. Clin Epigenetics.

[13] Moylan CA, Pang H, Dellinger A, Suzuki A, Garrett ME, Guy CD (2014). Hepatic gene expression profiles differentiate presymptomatic patients with mild versus severe nonalcoholic fatty liver disease. Hepatology.

[14] Anstee QM, Day CP (2015). The genetics of nonalcoholic fatty liver disease: spotlight on *PNPLA3* and *TM6SF2*. Semin Liver Dis.

[15] Kitamoto T, Kitamoto A, Yoneda M, Hyogo H, Ochi H, Mizusawa S (2014). Targeted next-generation sequencing and fine linkage disequilibrium mapping reveals association of *PNPLA3* and *PARVB* with the severity of nonalcoholic fatty liver disease. J Hum Genet.

[16] Kitamoto T, Kitamoto A, Ogawa Y, Honda Y, Imajo K, Saito S (2015). Targeted-bisulfite sequence analysis of the methylation of CpG islands in genes encoding *PNPLA3*, *SAMM50*, and *PARVB* of patients with non-alcoholic fatty liver disease. J Hepatol.

[17] Orozco LD, Morselli M, Rubbi L, Guo W, Go J, Shi H (2015). Epigenome-wide association of liver methylation patterns and complex metabolic traits in mice. Cell Metab.

[18] Maher JJ (2011). New insights from rodent models of fatty liver disease. Antioxid Redox Signal.

[19] Verdelho Machado M, Michelotti GA, Xie G, Pereira De Almeida T, Boursier J, Bohnic B (2015). Mouse models of diet-induced nonalcoholic steatohepatitis reproduce the heterogeneity of the human disease. PLoS One.

[20] Tryndyak VP, Latendresse JR, Montgomery B, Ross SA, Beland FA, Rusyn I (2012). Plasma microRNAs are sensitive indicators of inter-strain differences in the severity of liver injury induced in mice by a choline- and folate-deficient diet. Toxicol Appl Pharmacol.

[21] Ernst J, Kheradpour P, Mikkelsen TS, Shoresh N, Ward LD, Epstein CB (2011). Mapping and analysis of chromatin state dynamics in nine human cell types. Nature.

[22] Tryndyak V, de Conti A, Kobets T, Kutanzi K, Koturbash I, Han T (2012). Interstrain differences in the severity of liver injury induced by a choline- and folate-deficient diet in mice are associated with dysregulation of genes involved in lipid metabolism. FASEB J.

[23] Chalasani N, Guo X, Loomba R, Goodarzi MO, Haritunians T, Kwon S (2010). Genome-wide association study identifies variants associated with histologic features of nonalcoholic fatty liver disease. Gastroenterology.

[24] Di Rosa M, Malaguarnera L (2012). Genetic variants in candidate genes influencing NAFLD progression. J Mol Med (Berl).

[25] Daly AK, Ballestri S, Carulli L, Loria P, Day CP (2011). Genetic determinants of susceptibility and severity in nonalcoholic fatty liver disease. Expert Rev Gastroenterol Hepatol.

[26] Hernaez R (2012). Genetic factors associated with the presence and progression of nonalcoholic fatty liver disease: a narrative review. Gastroenterol Hepatol.

[27] Sookoian S, Castaño GO, Scian R, Mallardi P, Fernández Gianotti T, Burgueño AL (2015). Genetic variation in transmembrane 6 superfamily member 2 and the risk of nonalcoholic fatty liver disease and histological disease severity. Hepatology.

[28] Pogribny IP, Tryndyak VP, Bagnyukova TV, Melnyk S, Montgomery B, Ross SA (2009). Hepatic epigenetic phenotype predermines individual susceptibility to hepatic steatosis in mice fed a lipogenic methyl-deficient diet. J Hepatol.

[29] Moya M, Benet M, Guzmán C, Tolosa L, García-Monzón C, Pareja E (2012). Foxa1 reduces lipid accumulation in human hepatocytes and is down-regulated in nonalcoholic fatty liver. PLoS One.

[30] Guzmán C, Benet M, Pisonero-Vaquero S, Moya M, García-Mediavilla MV, Martínez-Chantar ML (1831). The human liver fatty acid binding protein (FABP1) gene is activated by FOXA1 and PPARα; and repressed by C/EBPα: implications in FABP1 down-regulation in nonalcoholic fatty liver disease. Biochim Biophys Acta.

[31] Tsuchiya H, da Costa K-A, Lee S, Renga B, Jaeschke H, Yang Z (2015). Interactions between nuclear receptor SHP and FOXA1 maintain oscillatory homocysteine homeostasis in mice. Gastroenterology.

[32] Schierwagen R, Maybüchen L, Zimmer S, Hittatiya K, Bäck C, Klein S (2015). Seven weeks of Western diet in apolipoprotein-E-deficient mice induce metabolic syndrome and non-alcoholic steatohepatitis with liver fibrosis. Sci Rep.

[33] Benet M, Moya M, Donato MT, Lahoz A, Hervás D, Guzmán C (2014). A simple transcriptomic signature able to predict drug-induced hepatic steatosis. Arch Toxicol.

[34] Fujimori K, Amano F (2011). Forkhead transcription factor Foxa1 is a novel target gene of C/EBPβ and suppresses the early phase of adipogenesis. Gene.

[35] Plumb-Rudewiez N, Clotman F, Strick-Marchand H, Pierreux CE, Weiss MC, Rousseau GG (2004). Transcription factor HNF-6/OC-1 inhibits the stimulation of the *HNF-3α/Foxa1* gene by TGF-β in mouse liver. Hepatology.

[36] Deng X-G, Qiu R-L, Wu Y-H, Li Z-X, Xie P, Zhang J (2014). Overexpression of miR-122 promotes the hepatic differentiation and maturation of mouse ESCs through a miR-122/FoxA1/HNF4a-positive feedback loop. Liver Int.

[37] Fang H, Harris SC, Su Z, Chen M, Qian F, Shi L (2009). ArrayTrack: an FDA and public genomic tool. Methods Mol Biol.

[38] Schmittgen TD, Livak KJ (2008). Analyzing real-time PCR data by the comparative *C*_T_ method. Nat Protoc.

