# Supplementary material for: Status of hepatic DNA methylome predetermines and modulates the severity of non-alcoholic fatty liver injury in mice
Source: BMC Genomics. 2016 Apr 22;17:298. doi: 10.1186/s12864-016-2617-2 (PMC4840954; doi:10.1186/s12864-016-2617-2)
Supplement: Additional file 1: Table S1. — Differentially methylated CpG island (CGI) in promoters of differentially expressed genes in the livers of WSB/EiJ mice fed a CFD diet for 12 weeks. (DOC 298 kb) [file 12864_2016_2617_MOESM1_ESM.doc]

**Supplementary Table 1. Differentially methylated CpG island (CGI) in promoters of differentially expressed genes in the livers of WSB/EiJ mice fed a CFD diet for 12 weeks.**

| **#** | **CGI chromosome location** | **Gene name** | **Gene description** | **MeDIP**  **CFD diet vs Control** | | **Gene expression**  **CFD diet vs Control** | |
| --- | --- | --- | --- | --- | --- | --- | --- |
|  |  |  |  | **Fold change** | **p value** | **Fold change** | **p value** |
| 1. 1 | chr15:31297620-31297664 | Ankrd33b | Ankyrin repeat domain 33B | 31.91 | 0.005 | -2.94 | 0.001 |
|  | chr15:31297506-31297550 | Ankrd33b | Ankyrin repeat domain 33B | 24.95 | 0.009 | -2.94 | 0.001 |
|  | chr2:147869669-147869713 | Foxa2 | Forkhead box a2 | 23.06 | 0.001 | -2.33 | 0.013 |
|  | chr15:58914108-58914152 | Mtss1 | Metastasis suppressor 1 | 20.58 | 0.006 | -2.68 | < 0.001 |
|  | chr12:113960025-113960073 | AW555464 | Expressed sequence AW555464 | 18.63 | 0.008 | -4.26 | < 0.001 |
|  | chr2:168427374-168427425 | Nfatc2 | Nuclear factor of activated T-cells, cytoplasmic, calcineurin-dependent 2 | 12.35 | 0.020 | -2.90 | < 0.001 |
|  | chr7:121706623-121706670 | Cyp2r1 | Cytochrome P450, family 2, subfamily r, polypeptide 1 | 11.64 | 0.006 | -2.56 | < 0.001 |
|  | chr1:75216487-75216531 | Tuba4a | Tubulin, alpha 4A | 11.16 | 0.011 | -3.12 | < 0.001 |
|  | chr15:31297783-31297827 | Ankrd33b | Ankyrin repeat domain 33B | 10.54 | 0.021 | -2.94 | 0.001 |
|  | chr15:89204625-89204669 | Sco2 | SCO cytochrome oxidase deficient homolog 2 | 10.39 | 0.015 | -2.78 | 0.001 |
|  | chr12:111517314-111517358 | Dio3 | Deiodinase, iodothyronine type III | 10.38 | 0.006 | -2.06 | 0.039 |
|  | chr13:55815861-55815913 | Txndc15 | Thioredoxin domain containing 15 | 9.75 | 0.009 | -2.95 | < 0.001 |
|  | chr4:43644731-43644775 | Npr2 | Natriuretic peptide receptor 2 | 9.66 | 0.018 | -2.68 | 0.001 |
|  | chr10:79811490-79811534 | Adamtsl5 | ADAMTS-like 5 | 9.09 | 0.007 | -2.04 | 0.003 |
|  | chr15:79763884-79763928 | Cbx7 | Chromobox protein homolog 7 | 8.79 | 0.016 | -2.30 | 0.004 |
|  | chr15:79763967-79764012 | Cbx7 | Chromobox protein homolog 7 | 8.51 | 0.002 | -2.30 | 0.004 |
|  | chr12:113960182-113960226 | AW555464 | Expressed sequence AW555464 | 8.36 | 0.026 | -4.26 | < 0.001 |
|  | chr10:114238616-114238660 | Trhde | TRH-degrading enzyme | 8.20 | 0.024 | -30.86 | < 0.001 |
|  | chr11:78279060-78279104 | Slc46a1 | Solute carrier family 46, member 1 | 8.18 | 0.008 | -2.22 | < 0.001 |
|  | chr18:74427538-74427582 | Mbd1 | Methyl-cpg binding domain protein 1 | 8.13 | 0.009 | -3.45 | 0.003 |
|  | chr10:79811706-79811750 | Adamtsl5 | ADAMTS-like 5 | 7.91 | 0.006 | -2.04 | 0.003 |
|  | chr12:111517382-111517429 | Dio3 | Deiodinase, iodothyronine type III | 7.79 | 0.029 | -2.06 | 0.039 |
|  | chr17:34165061-34165105 | H2-Ke6 | H2-K region expressed gene 6 | 7.71 | 0.008 | -3.01 | < 0.001 |
|  | chr11:95685266-95685311 | Phospho1 | Phosphatase, orphan 1 | 7.68 | 0.008 | -2.67 | < 0.001 |
|  | chr11:70796668-70796715 | C1qbp | Complement component 1, q subcomponent binding protein | 7.58 | 0.007 | -2.29 | < 0.001 |
|  | chr15:58913796-58913840 | Mtss1 | Metastasis suppressor 1 | 7.50 | 0.001 | -2.68 | < 0.001 |
|  | chr2:168427472-168427516 | Nfatc2 | Nuclear factor of activated T-cells, cytoplasmic, calcineurin-dependent 2 | 7.31 | 0.014 | -2.90 | < 0.001 |
|  | chr5:31223704-31223748 | Khk | Ketohexokinase | 7.17 | 0.030 | -2.02 | < 0.001 |
|  | chr2:168427638-168427682 | Nfatc2 | Nuclear factor of activated T-cells, cytoplasmic, calcineurin-dependent 2 | 7.16 | 0.031 | -2.90 | < 0.001 |
|  | chr4:150228926-150228970 | Errfi1 | ERBB receptor feedback inhibitor 1 | 7.00 | 0.026 | -2.47 | < 0.001 |
|  | chr5:31223835-31223879 | Khk | Ketohexokinase | 6.96 | 0.023 | -2.02 | < 0.001 |
|  | chr5:35731675-35731719 | Hmx1 | H6 homeobox 1 | 6.77 | 0.016 | -2.51 | 0.023 |
|  | chr7:20281867-20281911 | Apoe | Apolipoprotein E | 6.66 | 0.049 | -2.11 | < 0.001 |
|  | chr12:28027843-28027887 | Sox11 | SRY-box containing gene 11 | 6.42 | 0.017 | -2.78 | 0.007 |
|  | chr12:113960100-113960144 | AW555464 | Expressed sequence AW555464 | 6.33 | 0.039 | -4.26 | < 0.001 |
|  | chr1:75216331-75216375 | Tuba4a | Tubulin, alpha 4A | 6.29 | 0.021 | -3.12 | < 0.001 |
|  | chr5:108863529-108863582 | Atp5k | ATP synthase, H+ transporting, mitochondrial F1F0 complex, subunit e | 6.20 | 0.042 | -2.39 | < 0.001 |
|  | chr14:31528058-31528108 | Sfmbt1 | Scm-like with four mbt domains 1 | 6.12 | 0.006 | -2.22 | < 0.001 |
|  | chr4:136018363-136018407 | Luzp1 | Leucine zipper protein 1 | 6.08 | 0.010 | -2.31 | 0.004 |
|  | chr16:4419704-4419749 | Adcy9 | Adenylate cyclase 9 | 6.08 | 0.044 | -2.71 | 0.001 |
|  | chr13:55815959-55816003 | Txndc15 | Thioredoxin domain containing 15 | 5.93 | 0.007 | -2.95 | < 0.001 |
|  | chr4:84530030-84530074 | Cntln | Centlein, centrosomal protein | 5.91 | 0.013 | -3.10 | < 0.001 |
|  | chr12:58643471-58643515 | Foxa1 | Forkhead box a1 | 5.90 | 0.007 | -3.13 | 0.006 |
|  | chr16:62786539-62786583 | Nsun3 | NOL1/NOP2/Sun domain family member 3 | 5.77 | 0.034 | -2.42 | 0.019 |
|  | chr16:62786784-62786833 | Nsun3 | NOL1/NOP2/Sun domain family member 3 | 5.58 | 0.000 | -2.42 | 0.019 |
|  | chr17:46866138-46866182 | Gnmt | Glycine N-methyltransferase | 5.48 | 0.029 | -4.62 | 0.001 |
|  | chr18:61195926-61195970 | Cdx1 | Caudal type homeobox 1 | 5.36 | 0.020 | -4.20 | 0.027 |
|  | chr6:90412231-90412286 | Klf15 | Kruppel-like factor 15 | 5.20 | 0.019 | -2.50 | 0.006 |
|  | chr8:112692274-112692318 | Calb2 | Calbindin 2 | 5.18 | 0.033 | -2.51 | 0.034 |
|  | chr14:31528289-31528333 | Sfmbt1 | Scm-like with four mbt domains 1 | 5.14 | 0.033 | -2.22 | < 0.001 |
|  | chr3:94965500-94965544 | Sema6c | Sema domain, transmembrane domain (TM), and cytoplasmic domain, (semaphorin) 6C | 5.12 | 0.035 | -2.01 | 0.006 |
|  | chr11:11937756-11937805 | Grb10 | Growth factor receptor bound protein 10 | 4.96 | 0.024 | -4.73 | < 0.001 |
|  | chr12:88489043-88489093 | Pomt2 | Protein-O-mannosyltransferase 2 | 4.95 | 0.031 | -3.10 | < 0.001 |
|  | chr15:89204542-89204586 | Sco2 | SCO cytochrome oxidase deficient homolog 2 | 4.95 | 0.022 | -2.78 | 0.001 |
|  | chr16:9995375-9995419 | Grin2a | Glutamate receptor, ionotropic, NMDA2A (epsilon 1) | 4.93 | 0.030 | -2.21 | 0.008 |
|  | chr8:87308180-87308224 | Nfix | Nuclear factor I/X | 4.93 | 0.030 | -2.58 | < 0.001 |
|  | chr7:31742709-31742753 | Usf2 | Upstream transcription factor 2 | 4.87 | 0.042 | -2.21 | < 0.001 |
|  | chr16:17132713-17132758 | Sdf2l1 | Stromal cell-derived factor 2-like 1 | 4.83 | 0.016 | -2.24 | 0.002 |
|  | chr15:84962496-84962540 | Ribc2 | RIB43A domain with coiled-coils 2 | 4.62 | 0.030 | -2.08 | 0.011 |
|  | chr2:31492494-31492545 | Prdm12 | PR domain containing 12 | 4.60 | 0.033 | -2.13 | 0.021 |
|  | chr8:87308048-87308097 | Nfix | Nuclear factor I/X | 4.56 | 0.024 | -2.58 | < 0.001 |
|  | chr2:147014006-147014062 | Nkx2-2 | NK2 transcription factor related, locus 2 | 4.52 | 0.031 | -2.32 | 0.013 |
|  | chr10:79811999-79812043 | Adamtsl5 | ADAMTS-like 5 | 4.40 | 0.023 | -2.04 | 0.003 |
|  | chr10:114238703-114238747 | Trhde | TRH-degrading enzyme | 4.38 | 0.041 | -30.86 | < 0.001 |
|  | chr3:129581331-129581384 | Pla2g12a | Phospholipase A2, group XIIA | 4.38 | 0.008 | -2.09 | < 0.001 |
|  | chr12:109514810-109514861 | Ccdc85c | Coiled-coil domain containing 85C | 4.35 | 0.050 | -2.49 | 0.028 |
|  | chr19:36811512-36811563 | Ppp1r3c | Protein phosphatase 1, regulatory (inhibitor) subunit 3C | 4.33 | 0.011 | -5.59 | < 0.001 |
|  | chr11:95937358-95937402 | Atp5g1 | ATP synthase, H+ transporting, mitochondrial F0 complex, subunit c1 (subunit 9) | 4.33 | 0.000 | -2.17 | < 0.001 |
|  | chr11:80966386-80966430 | Accn1 | Amiloride-sensitive cation channel 1, neuronal | 4.31 | 0.018 | -2.05 | 0.014 |
|  | chr2:74497639-74497686 | Evx2 | Even skipped homeotic gene 2 homolog | 4.26 | 0.014 | -2.02 | 0.011 |
|  | chr19:23210880-23210927 | Klf9 | Kruppel-like factor 9 | 4.25 | 0.034 | -2.10 | 0.003 |
|  | chr2:32549720-32549764 | Fpgs | Folylpolyglutamyl synthetase | 4.20 | 0.024 | -2.64 | < 0.001 |
|  | chr11:33743828-33743872 | Kcnip1 | Kv channel-interacting protein 1 | 4.18 | 0.044 | -2.35 | 0.013 |
|  | chr5:108863393-108863437 | Atp5k | ATP synthase, H+ transporting, mitochondrial F1F0 complex, subunit e | 4.14 | 0.027 | -2.39 | < 0.001 |
|  | chr9:106062383-106062432 | Glyctk | Glycerate kinase | 4.13 | 0.008 | -2.14 | < 0.001 |
|  | chr14:31528182-31528230 | Sfmbt1 | Scm-like with four mbt domains 1 | 4.12 | 0.013 | -2.22 | < 0.001 |
|  | chr16:21204696-21204740 | Ephb3 | Eph receptor B3 | 4.11 | 0.042 | -4.78 | < 0.001 |
|  | chr6:112439818-112439862 | Oxtr | Oxytocin receptor | 4.06 | 0.049 | -2.71 | 0.002 |
|  | chr13:81850332-81850377 | Mblac2 | Metallo-beta-lactamase domain containing 2 | 4.06 | 0.049 | -2.58 | 0.001 |
|  | chr11:97547153-97547197 | Cisd3 | CDGSH iron sulfur domain 3 | 4.05 | 0.031 | -3.10 | < 0.001 |
|  | chr6:88674155-88674204 | Mgll | Monoglyceride lipase | 4.01 | 0.010 | -2.35 | 0.001 |
|  | chr2:49642849-49642898 | Lypd6b | LY6/PLAUR domain containing 6B | 3.96 | 0.006 | -2.29 | 0.037 |
|  | chr14:14844919-14844966 | Atxn7 | Ataxin 7 | 3.92 | 0.024 | -5.35 | < 0.001 |
|  | chr13:47110301-47110353 | Nhlrc1 | NHL repeat containing 1 | 3.90 | 0.040 | -2.16 | 0.049 |
|  | chr13:73466039-73466086 | Ndufs6 | NADH dehydrogenase (ubiquinone) 1 beta subcomplex 8 | 3.90 | 0.018 | -2.05 | < 0.001 |
|  | chr8:74967487-74967531 | Calr3 | Calreticulin 3 | 3.89 | 0.045 | -5.46 | < 0.001 |
|  | chr16:62786617-62786662 | Nsun3 | NOL1/NOP2/Sun domain family member 3 | 3.85 | 0.023 | -2.42 | 0.019 |
|  | chr13:40826354-40826399 | Tcfap2a | Transcription factor AP-2, alpha | 3.84 | 0.021 | -2.22 | 0.022 |
|  | chr1:136452840-136452886 | Kdm5b | Lysine (k)-specific demethylase 5b | 3.84 | 0.011 | -2.51 | 0.003 |
|  | chr18:58995914-58995958 | Adamts19 | A disintegrin-like and metallopeptidase (reprolysin type) with thrombospondin type 1 motif, 19 | 3.81 | 0.037 | -2.25 | 0.029 |
|  | chr10:79812076-79812120 | Adamtsl5 | ADAMTS-like 5 | 3.78 | 0.033 | -2.04 | 0.003 |
|  | chr3:129581715-129581759 | Pla2g12a | Phospholipase A2, group XIIA | 3.77 | 0.020 | -2.09 | < 0.001 |
|  | chr6:90412347-90412391 | Klf15 | Kruppel-like factor 15 | 3.73 | 0.024 | -2.50 | 0.006 |
|  | chr10:127630974-127631033 | Gls2 | Glutaminase 2 (liver, mitochondrial) | 3.66 | 0.008 | -3.88 | 0.002 |
|  | chr9:65477446-65477490 | Rbpms2 | RNA binding protein with multiple splicing 2 | 3.64 | 0.039 | -2.94 | < 0.001 |
|  | chr11:120479236-120479280 | Pcyt2 | Phosphate cytidylyltransferase 2, ethanolamine | 3.64 | 0.020 | -2.03 | < 0.001 |
|  | chr10:114799443-114799487 | Tmem19 | Transmembrane protein 19 | 3.60 | 0.043 | -2.09 | 0.001 |
|  | chr12:88331320-88331364 | Zdhhc22 | Zinc finger, DHHC-type containing 22 | 3.59 | 0.050 | -3.78 | < 0.001 |
|  | chr1:75216148-75216195 | Tuba4a | Tubulin, alpha 4A | 3.57 | 0.040 | -3.12 | < 0.001 |
|  | chr10:81063724-81063768 | Tle6 | Transducin-like enhancer of split 6 | 3.55 | 0.020 | -2.10 | 0.001 |
|  | chr3:104765067-104765111 | Wnt2b | Wingless related MMTV integration site 2b | 3.55 | 0.047 | -2.34 | 0.014 |
|  | chr5:140017148-140017192 | Uncx | UNC homeobox | 3.55 | 0.021 | -2.72 | 0.008 |
|  | chr11:90252510-90252569 | Hlf | Hepatic leukemia factor | 3.55 | 0.045 | -4.43 | < 0.001 |
|  | chr15:80502053-80502097 | Fam83f | Family with sequence similarity 83, member F | 3.54 | 0.028 | -4.15 | 0.001 |
|  | chr13:114584476-114584520 | Arl15 | ADP-ribosylation factor-like 15 | 3.48 | 0.009 | -2.44 | 0.001 |
|  | chr11:120522293-120522337 | Notum | Notum pectinacetylesterase homolog | 3.47 | 0.029 | -2.72 | 0.003 |
|  | chr3:129581524-129581568 | Pla2g12a | Phospholipase A2, group XIIA | 3.47 | 0.013 | -2.09 | < 0.001 |
|  | chr6:88674283-88674327 | Mgll | Monoglyceride lipase | 3.46 | 0.044 | -2.35 | 0.001 |
|  | chr14:34260620-34260664 | Mapk8 | Mitogen-activated protein kinase 8 | 3.42 | 0.039 | -2.13 | 0.024 |
|  | chr13:40833476-40833520 | Tcfap2a | Transcription factor AP-2, alpha | 3.38 | 0.008 | -2.22 | 0.022 |
|  | chr10:41998368-41998412 | Foxo3 | Forkhead box O3 | 3.37 | 0.017 | -2.38 | 0.006 |
|  | chr13:72101391-72101435 | Irx1 | Iroquois related homeobox 1 | 3.36 | 0.031 | -20.28 | 0.001 |
|  | chr2:26789394-26789445 | Gm711 | Predicted gene 711 | 3.36 | 0.026 | -2.41 | 0.006 |
|  | chr17:79420602-79420646 | Prkd3 | Protein kinase D3 | 3.35 | 0.028 | -2.88 | < 0.001 |
|  | chr10:79811365-79811409 | Adamtsl5 | ADAMTS-like 5 | 3.33 | 0.040 | -2.04 | 0.003 |
|  | chr10:79811178-79811222 | Adamtsl5 | ADAMTS-like 5 | 3.29 | 0.014 | -2.04 | 0.003 |
|  | chr2:31492078-31492122 | Prdm12 | PR domain containing 12 | 3.29 | 0.011 | -2.13 | 0.021 |
|  | chr3:63768819-63768878 | Slc33a1 | Solute carrier family 33 (acetyl-coa transporter), member 1 | 3.28 | 0.039 | -2.21 | < 0.001 |
|  | chr11:84642731-84642775 | Dhrs11 | Dehydrogenase/reductase (SDR family) member 11 | 3.27 | 0.027 | -2.72 | < 0.001 |
|  | chr4:46356192-46356241 | Foxe1 | Forkhead box E1 | 3.27 | 0.022 | -3.42 | 0.006 |
|  | chr2:32549787-32549832 | Fpgs | Folylpolyglutamyl synthetase | 3.23 | 0.028 | -2.64 | 0.000 |
|  | chrX:7215563-7215608 | Syp | Synaptophysin | 3.22 | 0.045 | -2.36 | 0.004 |
|  | chr3:40753323-40753367 | Larp1b | La ribonucleoprotein domain family, member 1B | 3.21 | 0.042 | -2.20 | 0.000 |
|  | chr5:136164752-136164796 | Por | P450 (cytochrome) oxidoreductase | 3.21 | 0.045 | -2.68 | 0.010 |
|  | chr6:89313162-89313207 | Plxna1 | Plexin A1 | 3.20 | 0.030 | -2.34 | 0.004 |
|  | chr3:94965569-94965620 | Sema6c | Sema domain, transmembrane domain (TM), and cytoplasmic domain, (semaphorin) 6C | 3.19 | 0.029 | -2.01 | 0.006 |
|  | chr18:74427757-74427801 | Mbd1 | Methyl-cpg binding domain protein 1 | 3.18 | 0.011 | -3.45 | 0.003 |
|  | chr2:105515679-105515724 | Pax6 | Paired box gene 6 | 3.18 | 0.033 | -2.00 | 0.016 |
|  | chr15:61869430-61869474 | Pvt1 | Plasmacytoma variant translocation 1 | 3.11 | 0.028 | -3.48 | < 0.001 |
|  | chr7:30074297-30074341 | Ppp1r14a | Protein phosphatase 1, regulatory (inhibitor) subunit 14A | 3.10 | 0.040 | -2.49 | < 0.001 |
|  | chr11:120522596-120522640 | Notum | Notum pectinacetylesterase homolog | 3.08 | 0.037 | -2.72 | 0.003 |
|  | chr11:103993774-103993818 | Crhr1 | Corticotropin releasing hormone receptor 1 | 3.08 | 0.043 | -2.05 | 0.027 |
|  | chr10:41998254-41998298 | Foxo3 | Forkhead box O3 | 3.05 | 0.046 | -2.38 | 0.006 |
|  | chr15:88785176-88785220 | Ttll8 | Tubulin tyrosine ligase-like family, member 8 | 3.03 | 0.027 | -2.09 | 0.019 |
|  | chr15:85565281-85565325 | Ppara | Peroxisome proliferator activated receptor alpha | 3.02 | 0.040 | -2.35 | < 0.001 |
|  | chr4:46356845-46356889 | Foxe1 | Forkhead box E1 | 2.98 | 0.017 | -3.42 | 0.006 |
|  | chr8:124103075-124103126 | Fbxo31 | F-box protein 31 | 2.98 | 0.031 | -2.84 | 0.000 |
|  | chr2:31495509-31495553 | Prdm12 | PR domain containing 12 | 2.97 | 0.023 | -2.13 | 0.021 |
|  | chr9:119071809-119071853 | Acaa1b | Acetyl-Coenzyme A acyltransferase 1B | 2.95 | 0.041 | -2.06 | 0.002 |
|  | chr10:127631429-127631473 | Gls2 | Glutaminase 2 (liver, mitochondrial) | 2.94 | 0.037 | -3.88 | 0.002 |
|  | chr1:133859504-133859548 | Slc45a3 | Solute carrier family 45, member 3 | 2.93 | 0.041 | -2.55 | 0.001 |
|  | chr10:126817352-126817400 | Inhbc | Inhibin beta-C | 2.92 | 0.025 | -2.09 | 0.007 |
|  | chr2:31491016-31491064 | Prdm12 | PR domain containing 12 | 2.91 | 0.005 | -2.13 | 0.021 |
|  | chr13:114584318-114584365 | Arl15 | ADP-ribosylation factor-like 15 | 2.89 | 0.043 | -2.44 | 0.001 |
|  | chr4:46663407-46663454 | Tbc1d2 | TBC1 domain family, member 2 | 2.89 | 0.044 | -2.66 | 0.001 |
|  | chr8:72839541-72839587 | Gdf1 | Growth differentiation factor 1 | 2.89 | 0.012 | -6.80 | 0.007 |
|  | chr10:74429603-74429649 | Gnaz | Guanine nucleotide binding protein, alpha z subunit | 2.84 | 0.019 | -2.36 | 0.008 |
|  | chr4:136392310-136392354 | Ephb2 | Eph receptor B2 | 2.83 | 0.035 | -4.26 | 0.001 |
|  | chr11:101177191-101177235 | Psme3 | Proteaseome | 2.82 | 0.028 | -5.61 | < 0.001 |
|  | chr11:51570336-51570395 | Sec24a | Sec24 related gene family, member A | 2.82 | 0.024 | -2.07 | < 0.001 |
|  | chr3:87723261-87723314 | Mrpl24 | Mitochondrial ribosomal protein L24 | 2.81 | 0.008 | -2.15 | < 0.001 |
|  | chr19:44629890-44629934 | Ndufb8 | NADH dehydrogenase (ubiquinone) 1 beta subcomplex 8 | 2.79 | 0.041 | -2.33 | < 0.001 |
|  | chr4:41688276-41688322 | Sigmar1 | Sigma non-opioid intracellular receptor 1 | 2.78 | 0.029 | -2.20 | < 0.001 |
|  | chr12:101348579-101348623 | Psmc1 | Protease | 2.77 | 0.033 | -2.16 | < 0.001 |
|  | chr7:39013316-39013360 | Plekhf1 | Pleckstrin homology domain containing, family F (with FYVE domain) member 1 | 2.72 | 0.037 | -2.03 | 0.002 |
|  | chr11:70796542-70796586 | C1qbp | Complement component 1, q subcomponent binding protein | 2.72 | 0.047 | -2.29 | < 0.001 |
|  | chr16:37868211-37868255 | Lrrc58 | Leucine rich repeat containing 58 | 2.69 | 0.049 | -2.08 | < 0.001 |
|  | chr17:46168375-46168419 | Vegfa | Vascular endothelial growth factor A | 2.69 | 0.038 | -2.10 | 0.001 |
|  | chr7:38556015-38556061 | Zfp536 | Zinc finger protein 536 | 2.64 | 0.044 | -10.09 | < 0.001 |
|  | chr14:13178482-13178526 | Fezf2 | Fez family zinc finger 2 | 2.63 | 0.048 | -2.08 | 0.014 |
|  | chr11:118884168-118884212 | Cbx2 | Chromobox homolog 2 | 2.63 | 0.022 | -2.91 | < 0.001 |
|  | chr10:79179060-79179104 | Hcn2 | Hyperpolarization-activated, cyclic nucleotide-gated K+ 2 | 2.63 | 0.013 | -2.15 | 0.009 |
|  | chr7:52744206-52744250 | Dhdh | Dihydrodiol dehydrogenase | 2.60 | 0.032 | -2.55 | 0.002 |
|  | chr3:63768695-63768739 | Slc33a1 | Solute carrier family 33 (acetyl-coa transporter), member 1 | 2.53 | 0.047 | -2.21 | < 0.001 |
|  | chr18:35658566-35658614 | Sil1 | Endoplasmic reticulum chaperone SIL1 homolog | 2.50 | 0.041 | -2.15 | < 0.001 |
|  | chr4:151382898-151382942 | Klhl21 | Kelch-like 21 | 2.48 | 0.024 | -2.25 | 0.002 |
|  | chr17:32326400-32326448 | Ephx3 | Epoxide hydrolase 3 | 2.47 | 0.049 | -2.00 | 0.023 |
|  | chr10:61595655-61595699 | Neurog3 | Neurogenin 3, mrna | 2.46 | 0.026 | -2.16 | 0.006 |
|  | chr5:118619079-118619130 | Hrk | Harakiri, BCL2 interacting protein (contains only BH3 domain) | 2.45 | 0.009 | -4.86 | 0.002 |
|  | chr2:49643006-49643050 | Lypd6b | LY6/PLAUR domain containing 6B | 2.42 | 0.048 | -2.29 | 0.037 |
|  | chr10:80381009-80381053 | Lmnb2 | Lamin B2 | 2.37 | 0.024 | -2.56 | 0.004 |
|  | chr3:104770737-104770781 | Wnt2b | Wingless related MMTV integration site 2b | 2.36 | 0.033 | -2.34 | 0.014 |
|  | chr11:79404418-79404462 | Rab11fip4 | RAB11 family interacting protein 4 (class II) | 2.36 | 0.029 | -2.43 | < 0.001 |
|  | chr13:40833642-40833688 | Tcfap2a | Transcription factor AP-2, alpha | 2.32 | 0.039 | -2.22 | 0.022 |
|  | chr19:47940093-47940137 | Gsto2 | Glutathione S-transferase omega 2 | 2.32 | 0.033 | -3.06 | 0.016 |
|  | chr2:69485168-69485213 | Bbs5 | Bardet-Biedl syndrome 5 | 2.31 | 0.030 | -2.90 | 0.000 |
|  | chr1:121734369-121734421 | Ptpn4 | Protein tyrosine phosphatase, non-receptor type 4 | 2.27 | 0.039 | -2.08 | 0.014 |
|  | chr4:46356459-46356503 | Foxe1 | Forkhead box E1 | 2.27 | 0.041 | -3.42 | 0.006 |
|  | chrX:96666163-96666209 | Pja1 | Praja1, RING-H2 motif containing | 2.26 | 0.014 | -2.07 | 0.000 |
|  | chr2:25733280-25733324 | Kcnt1 | Potassium channel, subfamily T, member 1 | 2.25 | 0.033 | -2.11 | 0.038 |
|  | chr5:35731082-35731126 | Hmx1 | H6 homeobox 1 | 2.23 | 0.041 | -2.51 | 0.023 |
|  | chr5:115879230-115879274 | Msi1 | Musashi homolog 1 | 2.17 | 0.010 | -2.16 | 0.003 |
|  | chr7:29221970-29222015 | Gmfg | Glia maturation factor, gamma | 2.14 | 0.036 | -2.03 | 0.022 |
|  | chr10:128111053-128111097 | Suox | Sulfite oxidase | 2.10 | 0.032 | -3.50 | 0.001 |
|  | chr1:136452733-136452792 | Kdm5b | Lysine (k)-specific demethylase 5b | 2.08 | 0.047 | -2.51 | 0.003 |
|  | chr6:88577556-88577600 | Kbtbd12 | Kelch repeat and BTB (POZ) domain containing 12 | 2.07 | 0.049 | -3.34 | 0.006 |
|  | chr6:48395590-48395634 | Zfp467 | Zinc finger protein 467 | 2.05 | 0.029 | -4.85 | < 0.001 |
|  | chr18:58995750-58995797 | Adamts19 | A disintegrin-like and metallopeptidase (reprolysin type) with thrombospondin type 1 motif, 19 | 2.01 | 0.029 | -2.25 | 0.029 |
|  | chr12:77923701-77923747 | Rab15 | RAB15, member RAS oncogene family | 2.00 | 0.044 | -2.06 | 0.009 |
|  | chr5:136220290-136220335 | Tmem120a | Transmembrane protein 120A | 1.99 | 0.023 | -2.20 | 0.001 |
|  | chr13:55254027-55254071 | Fgfr4 | Fibroblast growth factor receptor 4 | 1.97 | 0.046 | -2.02 | < 0.001 |
|  | chr10:61178081-61178125 | Aifm2 | Apoptosis-inducing factor, mitochondrion-associated 2 | 1.96 | 0.007 | -2.18 | 0.002 |
|  | chr2:91490251-91490295 | Arhgap1 | Rho gtpase activating protein 1 | 1.96 | 0.046 | -2.17 | 0.002 |
|  | chr4:134409828-134409872 | Tmem57 | Transmembrane protein 57 | 1.94 | 0.042 | -2.31 | < 0.001 |
|  | chr3:94965423-94965467 | Sema6c | Sema domain, transmembrane domain (TM), and cytoplasmic domain, (semaphorin) 6C | 1.92 | 0.043 | -2.01 | 0.006 |
|  | chr18:73914340-73914392 | Elac1 | Elac homolog 1 | 1.90 | 0.047 | -2.91 | 0.000 |
|  | chr4:124377609-124377660 | Fhl3 | Four and a half LIM domains 3 | 1.87 | 0.032 | -3.27 | 0.004 |
|  | chr1:133904124-133904168 | Elk4 | ELK4, member of ETS oncogene family | 1.76 | 0.048 | -2.27 | 0.001 |
|  | chr7:133990984-133991028 | Doc2a | Double C2, alpha | 1.75 | 0.007 | -2.15 | 0.010 |
|  | chr2:49642923-49642967 | Lypd6b | LY6/PLAUR domain containing 6B | 1.73 | 0.046 | -2.29 | 0.037 |
|  | chr15:76531943-76531987 | Mfsd3 | Major facilitator superfamily domain containing 3 | 1.72 | 0.040 | -2.44 | 0.002 |
|  | chr14:77556041-77556085 | Enox1 | Ecto-NOX disulfide-thiol exchanger 1 | 1.47 | 0.028 | -2.97 | 0.026 |
|  | chr10:79178951-79179001 | Hcn2 | Hyperpolarization-activated, cyclic nucleotide-gated K+ 2 | 1.42 | 0.037 | -2.15 | 0.009 |
|  | chr8:97442165-97442209 | Ccdc102a | Coiled-coil domain containing 102A | -1.30 | 0.045 | 2.61 | 0.005 |
|  | chr11:6428939-6428986 | Myo1g | Myosin IG | -1.46 | 0.049 | 14.56 | < 0.001 |
|  | chr12:114041031-114041075 | BC022687 | Cdna sequence BC022687 | -1.63 | 0.036 | 3.63 | 0.001 |
|  | chr4:109338163-109338221 | Cdkn2c | Cyclin-dependent kinase inhibitor 2C (p18, inhibits CDK4) | -1.77 | 0.043 | 2.27 | 0.001 |
|  | chr11:23979372-23979421 | Bcl11a | B-cell CLL/lymphoma 11A (zinc finger protein) | -1.79 | 0.049 | 5.07 | 0.022 |
|  | chr13:48721201-48721245 | Ptpdc1 | Protein tyrosine phosphatase domain containing 1 | -1.81 | 0.034 | 2.40 | 0.001 |
|  | chr1:60237015-60237065 | Nbeal1 | Neurobeachin like 1 | -1.89 | 0.035 | 2.12 | 0.010 |
|  | chr13:25147188-25147232 | Dcdc2a | Doublecortin domain containing 2a | -1.91 | 0.017 | 4.41 | 0.010 |
|  | chr9:77392817-77392861 | Lrrc1 | Leucine rich repeat containing 1 | -1.95 | 0.033 | 2.65 | 0.009 |
|  | chr9:31721571-31721630 | Barx2 | Barh-like homeobox 2 | -1.98 | 0.008 | 2.48 | 0.003 |
|  | chr13:50513180-50513224 | Fbxw17 | F-box and WD-40 domain protein 17 | -1.99 | 0.017 | 3.62 | < 0.001 |
|  | chr19:5875354-5875398 | Frmd8 | FERM domain containing 8 | -2.02 | 0.014 | 2.01 | < 0.001 |
|  | chr12:112081936-112081995 | Stk30 | Serine/threonine kinase 30 | -2.03 | 0.041 | 2.45 | < 0.001 |
|  | chr11:116392890-116392934 | Sphk1 | Sphingosine kinase 1 | -2.06 | 0.031 | 10.84 | 0.002 |
|  | chr13:15554596-15554640 | Gli3 | GLI-Kruppel family member GLI3 | -2.08 | 0.037 | 4.44 | 0.003 |
|  | chr11:68245484-68245528 | Pik3r5 | Phosphoinositide-3-kinase, regulatory subunit 5, p101 | -2.22 | 0.010 | 22.47 | 0.003 |
|  | chr7:31206709-31206753 | Nfkbid | Nuclear factor of kappa light polypeptide gene enhancer in B-cells inhibitor, delta | -2.27 | 0.008 | 3.67 | 0.001 |
|  | chr1:34063058-34063106 | Dst | Dystonin | -2.64 | 0.010 | 2.93 | 0.002 |
|  | chr5:121098665-121098712 | Rasal1 | RAS protein activator like 1 (GAP1 like) | -2.68 | 0.043 | 48.76 | < 0.001 |
|  | chr12:112082234-112082280 | Stk30 | Serine/threonine kinase 30 | -2.69 | 0.034 | 2.45 | < 0.001 |
|  | chr16:63864260-63864319 | Epha3 | Eph receptor A3 | -2.69 | 0.005 | 8.68 | 0.001 |
|  | chr10:80720218-80720262 | Matk | Megakaryocyte-associated tyrosine kinase | -2.72 | 0.035 | 3.12 | 0.003 |
|  | chr14:47993367-47993411 | Lgals3 | Lectin, galactose binding, soluble 3 | -2.77 | 0.031 | 57.85 | < 0.001 |
|  | chr17:43153334-43153378 | Tnfrsf21 | Tumor necrosis factor receptor superfamily, member 21 | -2.92 | 0.012 | 3.36 | 0.008 |
|  | chr3:14641575-14641619 | Car13 | Carbonic anhydrase 13 | -2.94 | 0.046 | 9.79 | 0.006 |
|  | chr14:47993280-47993326 | Lgals3 | Lectin, galactose binding, soluble 3 | -2.99 | 0.028 | 57.85 | < 0.001 |
|  | chr1:181597910-181597955 | Sccpdh | Saccharopine dehydrogenase (putative) | -2.99 | 0.017 | 3.29 | 0.001 |
|  | chr15:96473546-96473590 | Slc38a1 | Solute carrier family 38, member 1 | -3.04 | 0.005 | 4.39 | < 0.001 |
|  | chr7:26412259-26412311 | B3gnt8 | UDP-glcnac:betagal beta-1,3-N-acetylglucosaminyltransferase 8 | -3.09 | 0.043 | 9.33 | < 0.001 |
|  | chr11:116393075-116393119 | Sphk1 | Sphingosine kinase 1 | -3.09 | 0.006 | 10.84 | 0.002 |
|  | chr6:72908614-72908663 | Tmsb10 | Adult male small intestine cdna, RIKEN full-length enriched library, clone:2010309L16 product:thymosin, beta 10, full insert sequence. [AK008557] | -3.59 | 0.024 | 6.73 | < 0.001 |
|  | chr2:122124462-122124515 | Duoxa2 | Dual oxidase maturation factor 2 | -3.94 | 0.010 | 14.39 | < 0.001 |
|  | chr6:72908854-72908913 | Tmsb10 | Adult male small intestine cdna, RIKEN full-length enriched library, clone:2010309L16 product:thymosin, beta 10, full insert sequence. [AK008557] | -4.02 | 0.010 | 6.73 | < 0.001 |
|  | chr2:5058559-5058603 | Ccdc3 | Coiled-coil domain containing 3 | -4.28 | 0.044 | 11.69 | < 0.001 |
|  | chr13:93301246-93301303 | Zfyve16 | Zinc finger, FYVE domain containing 16 | -4.29 | 0.023 | 2.35 | < 0.001 |
|  | chr1:171461757-171461802 | Nuf2 | NUF2, NDC80 kinetochore complex component, homolog | -4.39 | 0.042 | 16.77 | < 0.001 |
|  | chr2:165329269-165329323 | Slc2a10 | Solute carrier family 2 (facilitated glucose transporter), member 10 | -4.40 | 0.011 | 4.79 | < 0.001 |
|  | chr8:120022048-120022092 | Plcg2 | Phospholipase C, gamma 2 | -4.60 | 0.027 | 6.75 | < 0.001 |
|  | chr9:31721645-31721701 | Barx2 | Barh-like homeobox 2 | -5.13 | 0.024 | 2.48 | 0.003 |
|  | chr2:165329453-165329497 | Slc2a10 | Solute carrier family 2 (facilitated glucose transporter), member 10 | -5.43 | 0.012 | 4.79 | < 0.001 |
|  | chr8:123649174-123649218 | Foxl1 | Forkhead box L1 | -49.43 | 0.032 | 2.13 | 0.033 |
