# Supplementary figures and images for: Status of hepatic DNA methylome predetermines and modulates the severity of non-alcoholic fatty liver injury in mice
Source: BMC Genomics. 2016 Apr 22;17:298. doi: 10.1186/s12864-016-2617-2 (PMC4840954; doi:10.1186/s12864-016-2617-2)

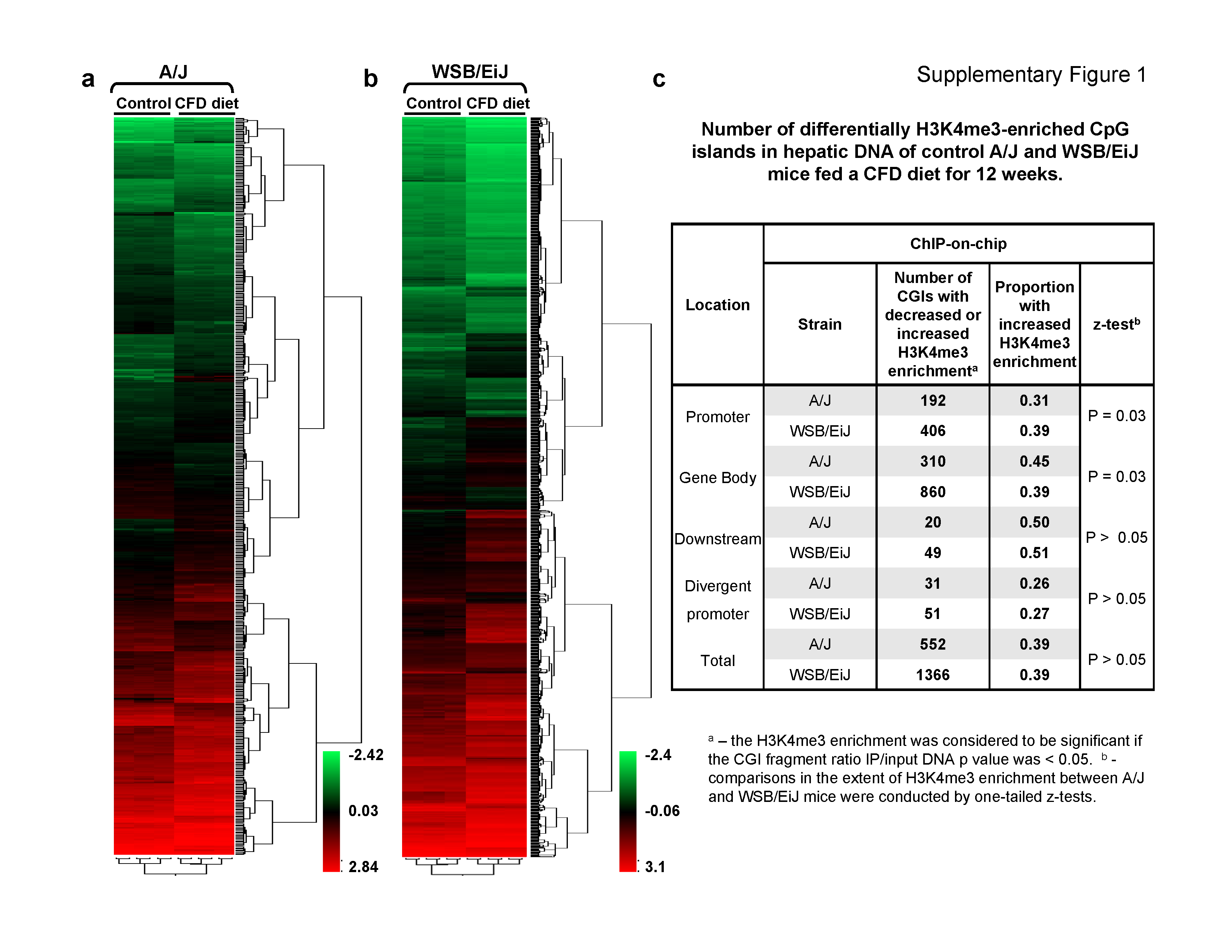

Supplement: Additional file 2: Figure S1. — H3K4me3 ChIP-on-chip analysis in the livers of A/J and WSB/EiJ mice fed a choline- and folate-deficient diet. Heat map illustrating significant differences in hepatic H3K4me3-enrichment of CpG islands between A/J (a) and WSB/EiJ (b) mice fed the CFD diet. Unsupervised hierarchical clustering analysis was performed using one-way ANOVA with p value cut-off at 0.05. The color bar identifies high-H3K4me3-enriched (red) and low-H3K4me3-enriched (green) genes. (c) Table showing number of differentially H3K4me3-enriched CpG islands in hepatic DNA in A/J and WSB/EiJ mice fed a control or CFD diet. Ratio IP/input DNA was calculated using ArrayTrack application. (TIFF 1501 kb) [file 12864_2016_2617_MOESM2_ESM.tiff]
